# Bis[2-(1*H*-benzotriazol-1-yl)acetonitrile-κ*N* ^3^]dibromidocopper(II)

**DOI:** 10.1107/S1600536808019260

**Published:** 2008-07-05

**Authors:** Wei Wang

**Affiliations:** a Ordered Matter Science Research Center, Southeast University, Nanjing 210096, People’s Republic of China

## Abstract

In the title complex, [CuBr_2_(C_8_H_6_N_4_)_2_], the Cu^II^ atom is located on an inversion centre and the asymmetric unit comprises one half-mol­ecule. The Cu atom is coordinated by two Br ions and two N atoms in approximately square-planar geometry. In the crystal structure, inter­molecular C—H⋯Br hydrogen bonds and π–π inter­actions between benzotriazole rings (centroid–centroid distance = 3.651 Å) generate a three-dimensional network.

## Related literature

For the synthesis of the organic ligand, see: Danan *et al*. (1997 ▶); Xu & Ye (2007 ▶). For the structure of a similar complex, see: Hang & Ye (2008 ▶).
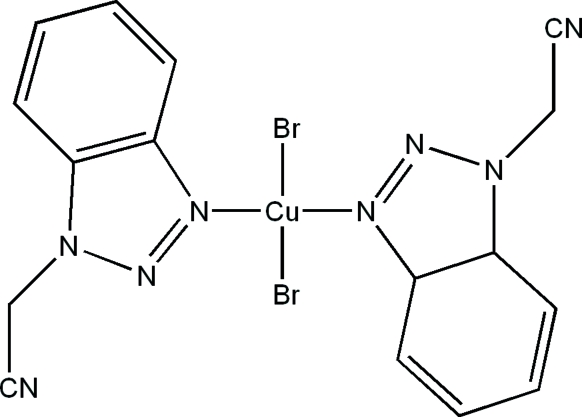


## Experimental

### 

#### Crystal data

[CuBr_2_(C_8_H_6_N_4_)_2_]
*M* *_r_* = 539.70
Triclinic, 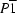

*a* = 7.9034 (16) Å
*b* = 8.1434 (16) Å
*c* = 8.7849 (18) Å
α = 116.04 (3)°
β = 105.86 (3)°
γ = 100.74 (3)°
*V* = 456.9 (3) Å^3^
*Z* = 1
Mo *K*α radiation
μ = 5.59 mm^−1^
*T* = 293 (2) K
0.20 × 0.12 × 0.12 mm

#### Data collection

Rigaku Mercury2 diffractometer
Absorption correction: multi-scan (*CrystalClear*; Rigaku, 2005 ▶) *T*_min_ = 0.702, *T*_max_ = 1.000 (expected range = 0.359–0.511)
4726 measured reflections
2095 independent reflections
1809 reflections with *I* > 2σ(*I*)
*R*_int_ = 0.037

#### Refinement

*R*[*F* ^2^ > 2σ(*F* ^2^)] = 0.067
*wR*(*F* ^2^) = 0.218
*S* = 1.06
2095 reflections
124 parameters
H-atom parameters constrained
Δρ_max_ = 3.13 e Å^−3^
Δρ_min_ = −1.39 e Å^−3^

### 

Data collection: *CrystalClear* (Rigaku, 2005 ▶); cell refinement: *CrystalClear*; data reduction: *CrystalClear*; program(s) used to solve structure: *SHELXS97* (Sheldrick, 2008 ▶); program(s) used to refine structure: *SHELXL97* (Sheldrick, 2008 ▶); molecular graphics: *SHELXTL* (Sheldrick, 2008 ▶); software used to prepare material for publication: *SHELXTL*.

## Supplementary Material

Crystal structure: contains datablocks I, global. DOI: 10.1107/S1600536808019260/kp2178sup1.cif

Structure factors: contains datablocks I. DOI: 10.1107/S1600536808019260/kp2178Isup2.hkl

Additional supplementary materials:  crystallographic information; 3D view; checkCIF report

## Figures and Tables

**Table:** 

Br1—Cu1	2.3385 (10)
Cu1—N3	2.012 (5)

**Table:** 

N3—Cu1—Br1	89.46 (16)

**Table 2:** Hydrogen-bond geometry (Å, °)

*D*—H⋯*A*	*D*—H	H⋯*A*	*D*⋯*A*	*D*—H⋯*A*
C7—H7*A*⋯Br1^i^	0.97	2.79	3.744 (7)	168
C7—H7*B*⋯Br1^ii^	0.97	2.91	3.421 (7)	114

Symmetry codes: (i) 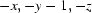; (ii) 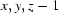.

**Fig. 1.**
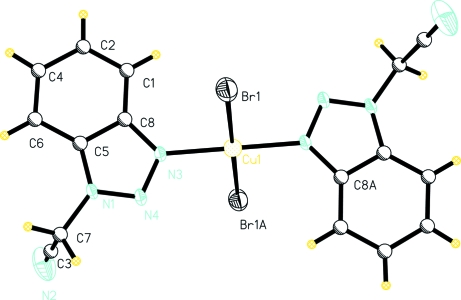
The molecular structure of the compound with the atomic numbering scheme. Displacement ellipsoids were drawn at the 30% probability level.

**Fig. 2.**
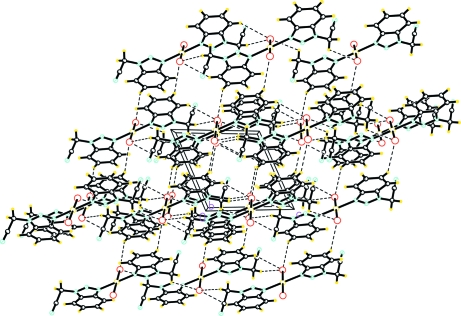
The crystal packing along b axis with π···π stacking.

## supplementary crystallographic information

### Comment

Recently, the crystal structure of 2-(1*H*-benzo[*d*][1,2,3] triazol-1-yl)acetonitrile (Xu *et al.* (2007)) and its Zn complex [Hang *et al.* (2008)] have been reported successively. Though adopting the same ligand, the structure of the title complex is quite different from the Zn analogue due to the different synthesis route. The precipitate of the title compound is obtained by mixing the ethanol solution of ligand and a water solution of CuBr_2_. Under this condition, the cyano group in the title compound does not hydrolyse nor coordinate to Cu^II^. Cu^II^ is coordinated by two nitrogen atoms from the benzotriazole rings and two terminal bromide anions in an almost square planar geometry (Fig. 1). The intermolecular C—H···Br hydrogen bonding interactions and π···π stacking between benzotriazole rings generate the three-dimensional network (Fig. 2); *Cg*···*Cg*^i^ distance is 3.651 Å, (symmetry code: -1-*x*,-1-y,-*z*; –*x*,-1-y,-*z*).

### Experimental

The ligand, 2-(1*H*-benzo[*d*][1,2,3]triazol-1-yl)acetonitrile, was synthesized by the reaction of benzotriazole and bromoacetonitrile according to the procedure described in the literature [Danan *et al.* (1997)].

2-(*1H*-benzotriazol-1-yl)acetonitrile (0.32 g,2 mmol) was dissolved in 5 mL ethanol, added into a solution of CuBr_2_(0.22 g,1 mmol) which was dissolved in 5 mL water, the mixture was filtered. Crystals suitable for X-ray analysis were obtained after standing the filtrate for 3 days at the room temperature.

### Refinement

Positional parameters of all the H atoms were calculated geometrically and were allowed to ride on the C, O atoms to which they are bonded, with *U*_iso_(H) = 1.2*U*_eq_(C) or *U*_iso_(H) = 1.5*U*_eq_(O).

### Crystal data

**Table:**

[CuBr_2_(C_8_H_6_N_4_)_2_]	*Z* = 1
*M**_r_* = 539.70	*F*_000_ = 263
Triclinic, *P*1	*D*_x_ = 1.961 Mg m^−^^3^
Hall symbol: -P 1	Mo *K*α radiation λ = 0.71073 Å
*a* = 7.9034 (16) Å	Cell parameters from 4665 reflections
*b* = 8.1434 (16) Å	θ = 6.2–57.6º
*c* = 8.7849 (18) Å	µ = 5.59 mm^−^^1^
α = 116.04 (3)º	*T* = 293 (2) K
β = 105.86 (3)º	Block, red
γ = 100.74 (3)º	0.20 × 0.12 × 0.12 mm
*V* = 456.9 (3) Å^3^	

### Data collection

**Table:**

Rigaku Mercury2 diffractometer	2095 independent reflections
Radiation source: fine-focus sealed tube	1809 reflections with *I* > 2σ(*I*)
Monochromator: graphite	*R*_int_ = 0.037
Detector resolution: 13.6612 pixels mm^-1^	θ_max_ = 27.5º
*T* = 293(2) K	θ_min_ = 3.1º
CCD_Profile_fitting scans	*h* = −10→10
Absorption correction: multi-scan(CrystalClear; Rigaku, 2005)	*k* = −10→10
*T*_min_ = 0.702, *T*_max_ = 1.000	*l* = −11→11
4726 measured reflections	

### Refinement

**Table:**

Refinement on *F*^2^	Secondary atom site location: difference Fourier map
Least-squares matrix: full	Hydrogen site location: inferred from neighbouring sites
*R*[*F*^2^ > 2σ(*F*^2^)] = 0.067	H-atom parameters constrained
*wR*(*F*^2^) = 0.218	*w* = 1/[σ^2^(*F*_o_^2^) + (0.1128*P*)^2^ + 5.2279*P*] where *P* = (*F*_o_^2^ + 2*F*_c_^2^)/3
*S* = 1.06	(Δ/σ)_max_ < 0.001
2095 reflections	Δρ_max_ = 3.13 e Å^−^^3^
124 parameters	Δρ_min_ = −1.39 e Å^−^^3^
Primary atom site location: structure-invariant direct methods	Extinction correction: none

### Special details

**Table:**

Geometry. All e.s.d.'s (except the e.s.d. in the dihedral angle between two l.s. planes) are estimated using the full covariance matrix. The cell e.s.d.'s are taken into account individually in the estimation of e.s.d.'s in distances, angles and torsion angles; correlations between e.s.d.'s in cell parameters are only used when they are defined by crystal symmetry. An approximate (isotropic) treatment of cell e.s.d.'s is used for estimating e.s.d.'s involving l.s. planes.
Refinement. Refinement of *F*^2^ against ALL reflections. The weighted *R*-factor *wR* and goodness of fit *S* are based on *F*^2^, conventional *R*-factors *R* are based on *F*, with *F* set to zero for negative *F*^2^. The threshold expression of *F*^2^ > 2σ(*F*^2^) is used only for calculating *R*-factors(gt) *etc*. and is not relevant to the choice of reflections for refinement. *R*-factors based on *F*^2^ are statistically about twice as large as those based on *F*, and *R*- factors based on ALL data will be even larger.

### Fractional atomic coordinates and isotropic or equivalent isotropic displacement parameters (Å^2^)

**Table:**

	*x*	*y*	*z*	*U*_iso_*/*U*_eq_	
Br1	0.13948 (13)	−0.20325 (12)	0.56625 (11)	0.0403 (3)	
Cu1	0.0000	0.0000	0.5000	0.0170 (3)	
C8	−0.2130 (9)	−0.4022 (9)	0.1349 (8)	0.0175 (12)	
N4	−0.0651 (9)	−0.1575 (8)	0.1166 (7)	0.0240 (12)	
N3	−0.1103 (9)	−0.2050 (8)	0.2291 (7)	0.0222 (12)	
N2	−0.3749 (15)	−0.2480 (17)	−0.3869 (13)	0.065 (3)	
C7	−0.1043 (11)	−0.3097 (11)	−0.2015 (9)	0.0242 (14)	
H7A	−0.0935	−0.4308	−0.2836	0.029*	
H7B	0.0128	−0.2042	−0.1526	0.029*	
C6	−0.3269 (10)	−0.6762 (10)	−0.1849 (9)	0.0258 (14)	
H6A	−0.3384	−0.7258	−0.3064	0.031*	
N1	−0.1353 (8)	−0.3207 (8)	−0.0490 (7)	0.0210 (11)	
C5	−0.2293 (9)	−0.4777 (9)	−0.0471 (8)	0.0181 (12)	
C4	−0.4036 (11)	−0.7908 (11)	−0.1270 (11)	0.0316 (16)	
H4A	−0.4675	−0.9237	−0.2119	0.038*	
C3	−0.2596 (12)	−0.2759 (12)	−0.3062 (10)	0.0325 (17)	
C2	−0.3899 (11)	−0.7159 (11)	0.0551 (11)	0.0297 (15)	
H2A	−0.4465	−0.8002	0.0862	0.036*	
C1	−0.2947 (10)	−0.5205 (10)	0.1897 (9)	0.0239 (13)	
H1A	−0.2860	−0.4712	0.3103	0.029*	

### Atomic displacement parameters (Å^2^)

**Table:**

	*U*^11^	*U*^22^	*U*^33^	*U*^12^	*U*^13^	*U*^23^
Br1	0.0515 (6)	0.0375 (5)	0.0321 (5)	0.0196 (4)	0.0162 (4)	0.0169 (4)
Cu1	0.0271 (6)	0.0117 (5)	0.0069 (5)	0.0051 (4)	0.0055 (4)	0.0022 (4)
C8	0.025 (3)	0.015 (3)	0.008 (3)	0.008 (2)	0.003 (2)	0.003 (2)
N4	0.038 (3)	0.017 (3)	0.011 (2)	0.008 (2)	0.008 (2)	0.005 (2)
N3	0.038 (3)	0.015 (3)	0.011 (2)	0.007 (2)	0.008 (2)	0.006 (2)
N2	0.075 (7)	0.088 (8)	0.037 (5)	0.052 (6)	0.018 (4)	0.031 (5)
C7	0.039 (4)	0.027 (3)	0.017 (3)	0.016 (3)	0.018 (3)	0.014 (3)
C6	0.029 (3)	0.020 (3)	0.012 (3)	0.009 (3)	0.004 (3)	−0.002 (2)
N1	0.035 (3)	0.017 (2)	0.010 (2)	0.010 (2)	0.009 (2)	0.006 (2)
C5	0.021 (3)	0.019 (3)	0.013 (3)	0.009 (2)	0.006 (2)	0.008 (2)
C4	0.030 (4)	0.017 (3)	0.032 (4)	0.004 (3)	0.006 (3)	0.005 (3)
C3	0.049 (5)	0.037 (4)	0.014 (3)	0.021 (4)	0.015 (3)	0.013 (3)
C2	0.028 (3)	0.024 (4)	0.033 (4)	0.006 (3)	0.010 (3)	0.015 (3)
C1	0.029 (3)	0.024 (3)	0.018 (3)	0.010 (3)	0.009 (3)	0.010 (3)

### Geometric parameters (Å, °)

**Table:**

Br1—Cu1	2.3385 (10)	C7—H7B	0.9700
Cu1—N3	2.012 (5)	C6—C4	1.368 (11)
C8—N3	1.379 (8)	C6—C5	1.407 (9)
C8—C1	1.387 (9)	C6—H6A	0.9300
C8—C5	1.395 (8)	N1—C5	1.363 (9)
N4—N3	1.316 (8)	C4—C2	1.402 (11)
N4—N1	1.333 (7)	C4—H4A	0.9300
N2—C3	1.118 (12)	C2—C1	1.383 (10)
C7—N1	1.462 (8)	C2—H2A	0.9300
C7—C3	1.470 (10)	C1—H1A	0.9300
C7—H7A	0.9700		
			
N3—Cu1—Br1	89.46 (16)	N4—N1—C5	111.7 (5)
N3—C8—C1	132.2 (6)	N4—N1—C7	118.6 (6)
N3—C8—C5	106.6 (6)	C5—N1—C7	129.7 (6)
C1—C8—C5	121.2 (6)	N1—C5—C8	104.5 (5)
N3—N4—N1	107.1 (5)	N1—C5—C6	132.8 (6)
N4—N3—C8	110.1 (5)	C8—C5—C6	122.7 (6)
N4—N3—Cu1	118.1 (4)	C6—C4—C2	122.7 (7)
C8—N3—Cu1	131.4 (4)	C6—C4—H4A	118.7
N1—C7—C3	111.1 (6)	C2—C4—H4A	118.7
N1—C7—H7A	109.4	N2—C3—C7	178.4 (10)
C3—C7—H7A	109.4	C1—C2—C4	122.0 (7)
N1—C7—H7B	109.4	C1—C2—H2A	119.0
C3—C7—H7B	109.4	C4—C2—H2A	119.0
H7A—C7—H7B	108.0	C2—C1—C8	116.3 (6)
C4—C6—C5	115.1 (6)	C2—C1—H1A	121.9
C4—C6—H6A	122.5	C8—C1—H1A	121.9
C5—C6—H6A	122.5		
			
N1—N4—N3—C8	−0.5 (8)	N4—N1—C5—C8	−0.4 (7)
N1—N4—N3—Cu1	172.6 (4)	C7—N1—C5—C8	179.9 (7)
C1—C8—N3—N4	−178.7 (7)	N4—N1—C5—C6	179.3 (7)
C5—C8—N3—N4	0.3 (8)	C7—N1—C5—C6	−0.5 (12)
C1—C8—N3—Cu1	9.4 (11)	N3—C8—C5—N1	0.0 (7)
C5—C8—N3—Cu1	−171.6 (5)	C1—C8—C5—N1	179.2 (6)
N3^i^—Cu1—N3—N4	−167 (92)	N3—C8—C5—C6	−179.7 (6)
Br1^i^—Cu1—N3—N4	59.4 (5)	C1—C8—C5—C6	−0.6 (10)
Br1—Cu1—N3—N4	−120.6 (5)	C4—C6—C5—N1	179.8 (7)
N3^i^—Cu1—N3—C8	4(93)	C4—C6—C5—C8	−0.5 (10)
Br1^i^—Cu1—N3—C8	−129.2 (6)	C5—C6—C4—C2	1.3 (11)
Br1—Cu1—N3—C8	50.8 (6)	N1—C7—C3—N2	150 (33)
N3—N4—N1—C5	0.5 (8)	C6—C4—C2—C1	−1.0 (12)
N3—N4—N1—C7	−179.7 (6)	C4—C2—C1—C8	−0.1 (11)
C3—C7—N1—N4	−91.8 (8)	N3—C8—C1—C2	179.7 (7)
C3—C7—N1—C5	87.9 (9)	C5—C8—C1—C2	0.9 (10)

Symmetry codes: (i) −*x*, −*y*, −*z*+1.

### Hydrogen-bond geometry (Å, °)

**Table:**

*D*—H···*A*	*D*—H	H···*A*	*D*···*A*	*D*—H···*A*
C7—H7A···Br1^ii^	0.97	2.79	3.744 (7)	168
C7—H7B···Br1^iii^	0.97	2.91	3.421 (7)	114

Symmetry codes: (ii) −*x*, −*y*−1, −*z*; (iii) *x*, *y*, *z*−1.
